# Magnetic-Responsive Liposomal Hydrogel Membranes for Controlled Release of Small Bioactive Molecules—An Insight into the Release Kinetics

**DOI:** 10.3390/membranes13070674

**Published:** 2023-07-17

**Authors:** Luís Pereira, Frederico Castelo Ferreira, Filipa Pires, Carla A. M. Portugal

**Affiliations:** 1 LAQV-REQUIMTE, Department of Chemistry, NOVA School of Science and Technology (FCT NOVA), Universidade Nova de Lisboa, 2829-516 Caparica, Portugal; lfc.pereira@campus.fct.unl.pt; 2 Department of Bioengineering and iBB—Institute for Bioengineering and Biosciences, Instituto Superior Técnico, Universidade de Lisboa, 1049-001 Lisboa, Portugal; frederico.ferreira@ist.utl.pt; 3 Associate Laboratory i4HB—Institute for Health and Bioeconomy, Instituto Superior Técnico, Universidade de Lisboa, Av. Rovisco Pais, 1049-001 Lisboa, Portugal

**Keywords:** drug release kinetics, magnetic field, liposomes

## Abstract

This work explores the unique features of magnetic-responsive hydrogels to obtain liposomal hydrogel delivery platforms capable of precise magnetically modulated drug release based on the mechanical responses of these hydrogels when exposed to an external magnetic field. Magnetic-responsive liposomal hydrogel delivery systems were prepared by encapsulation of 1,2-dipalmitoyl-sn-glycero-3-phosphocoline (DPPC) multilayered vesicles (MLVs) loaded with ferulic acid (FA), i.e., DPPC:FA liposomes, into gelatin hydrogel membranes containing dispersed iron oxide nanoparticles (MNPs), i.e., magnetic-responsive gelatin. The FA release mechanisms and kinetics from magnetic-responsive liposomal gelatin were studied and compared with those obtained with conventional drug delivery systems, e.g., free liposomal suspensions and hydrogel matrices, to access the effect of liposome entrapment and magnetic field on FA delivery. FA release from liposomal gelatin membranes was well described by the Korsmeyer–Peppas model, indicating that FA release occurred under a controlled diffusional regime, with or without magnetic stimulation. DPPC:FA liposomal gelatin systems provided smoother controlled FA release, relative to that obtained with the liposome suspensions and with the hydrogel platforms, suggesting the promising application of liposomal hydrogel systems in longer-term therapeutics. The magnetic field, with low intensity (0.08 T), was found to stimulate the FA release from magnetic-responsive liposomal gelatin systems, increasing the release rates while shifting the FA release to a quasi-Fickian mechanism. The magnetic-responsive liposomal hydrogels developed in this work offer the possibility to magnetically activate drug release from these liposomal platforms based on a non-thermal related delivery strategy, paving the way for the development of novel and more efficient applications of MLVs and liposomal delivery systems in biomedicine.

## 1. Introduction

Conventional clinical approaches, consisting of the direct administration of therapeutic compounds, have shown limited treatment efficiency, attributed to the short lifetime of these compounds, to the loss of the therapeutic molecules caused by loose guidance to the point of action and to the poor bioavailability of these bioactive compounds due to their low solubility in aqueous media (such as the case of lipophilic molecules). These problems have been circumvented by the increase of the drug doses, which may, however, potentiate drug cytotoxicity and the possibility of undesirable side effects.

Advances in this topic have been attempted through the development of biomimetic drug carriers, such as liposomes [1,2,3,4,5,6,7,8], micelles [9,10,11,12,13] and hydrogels [14,15,16,17,18,19]. These drug vehicles are able to accommodate and protect the drug/bioactive molecules (e.g., growth factors, peptides, genes, vaccines, and cells) from hydrolytic or enzymatic degradation in the organism. However, they lack proper delivery efficiency given their structural instability [20] and consequent incapacity to hold the drug molecules, resulting in an uncontrolled drug release, e.g., burst release [21,22,23]. Efforts to develop delivery systems with improved stability have been carried out through the optimization of the chemical formulation of these delivery vehicles, mainly through the addition of structural stabilizers (e.g., cholesterol and poly-L-lysine) [24,25,26,27]. Cholesterol was found to enhance the lipophilic packing in the liposome bilayer, providing improved liposome stability [24], whereas the hydrophobic molecules/polymers have been added to strength bond interactions, providing improved liposome resistance to enzymatic or immune reactions [28,29]. Thus, a fine regulation of the permeability, fluidity and temperature transition of these vesicles is possible by rigorous control of the amount of the stabilizing component in the liposome structure. 

An alternative approach takes advantage of a synergetic combination of liposomal technologies and the versatility of hydrogels to obtain delivery systems- liposomal hydrogel systems, with improved stability which are able to offer an enhanced controlled drug release [30]. Liposomal hydrogels have been developed either by the immobilization of gels in the liposome core [31,32,33,34] or by the encapsulation of liposomes into the hydrogel network [35,36,37,38]. These systems provide a double advantage; on the one hand, hydrogels confer higher liposome stability and protection against pH and ionic strength [30,37]. On the other hand, the encapsulated liposomes act as drug pockets with enhanced stability, leading to a more sustained and prolonged drug release comparatively than that found for conventional liposomes [39,40,41,42] or native hydrogel matrices [25]. Liposomal hydrogel delivery systems have shown improved efficiency for the treatment of several inflammatory diseases [43] and chemotherapy [44,45] comparatively to conventional therapies, ascribed to their ability to provide longer-term and in situ release of the target therapeutic compound, minimizing the toxic side effects due to drug leakage.

The improved stability of liposomal hydrogel systems results from a symbiotic effect that combines enhanced structural liposome stability conferred by the interaction with the hydrogel matrix, but, also, liposomes were found to act as stabilizing elements providing liposomal hydrogel systems with improved viscoelastic properties, conferring them additional structural cohesion [46,47].

The release kinetics and mechanisms from liposomal hydrogels are thus dependent on the combined transport resistance of the liposomal lipidic bilayer and the polymer matrix, which creates an additional rate-limiting effect on the diffusion of the therapeutic molecules. In this respect, the drug release is regulated by the hydrogel matrix’s structural and chemical properties (e.g., density, porosity and hydrophilicity) and by the permeability of the liposome membrane to the target drug. In fact, studies from other authors have also shown that the transport from liposomal hydrogels is importantly dependent on the liposome characteristics, i.e., vesicle chemical formulation and surface charge [48], and drug hydrophilicity. As reported by Mourtas et al. [36], the release of lipophilic molecules is predominantly influenced by the drug properties and loading, in contrast to that observed for hydrophilic drugs whose diffusion from liposomal hydrogels depends on the characteristics of the bilipid vesicle, i.e., chemical formulation and rigidity.

The large structural and chemical versatility of the hydrogels as well as their ability to respond to external stimuli (e. g. temperature, light, magnetic field) have been also explored to obtain liposomal hydrogels offering more precise spatiotemporal controlled drug release. Magnetic-responsive liposomal hydrogels offer the possibility of a stimuli-triggered drug release, allowing for an on-demand diffusion of the drug/bioactive molecules in the site of action while avoiding drug losses during the trajectory of the delivery vehicle to the target tissue. Magnetic liposomal systems have been developed, relying on the hyperthermic effect produced by magnetic susceptible nanoparticles (e.g., iron oxide nanoparticles)—MNPs—when exposed to a high-frequency magnetic field (AMF) [49,50,51,52,53]. MNPs may be present in the aqueous liposome core, embedded in the lipidic bilayer or as a solid MNP cluster forming the inner liposome core [47]. In both cases, magnetic-induced thermal release is prompted by the phase transition of the hydrogel from a solid state to a sol state at a specific temperature [51,52,53].

However, magnetic-responsive hydrogels exhibit unique mechanical responses upon magnetic stimulation, which have been very weakly explored in the development of magnetic-responsive liposomal systems. Magnetic-responsive hydrogels can reversibly switch their volume and shape when exposed to external magnetic field stimuli [54,55,56]. Such mechanical elastic distortions are due to the mobility of MNPs, imprisoned in the polymer network, in response to the attractive magnetic field forces. The mechanical distortions of hydrogels can be converted into forces and potentially interact with the entrapped liposomes, prompting the magnetically controlled release of the target bioactive molecules due to the structural destabilization of these vesicles and/or changes in the permeability of the liposome membranes. Magnetically controlled delivery may be thus obtained based on the mobility of the MNPs triggered by permanent or low-frequency magnetic field stimuli exploring the mechanical actuation of magnetic-responsive hydrogels, without thermal effects and thus extending the application of magnetic liposomal systems to compounds showing lower resistance to higher temperatures.

The present study proposes the development of magnetic-responsive liposomal hydrogels, which may be able to provide a sustainable magnetically controlled drug release prompted by the mechanical responses of magnetic-responsive hydrogels when exposed to an external magnetic field and not based on local thermal changes resulting from hyperthermia effects. The liposomal hydrogels were prepared by encapsulation of 1,2-dipalmitoyl-sn-glycero-3-phosphocoline (DPPC) liposomes loaded with ferulic acid (FA) into a gelatin matrix containing dispersed MNPs. FA was selected as a representative model of small therapeutic molecules with poor bioavailability. Furthermore, the FA’s well-known anti-inflammatory and pro-angiogenic properties were also considered, envisaging the future application of these liposomal hydrogel systems in long-term therapeutics requiring sustained controlled drug release and/or as part of tissue engineering tools for enhanced tissue repair [57]. The first stage of this work is focused on the optimization of the DPPC:FA liposome formation procedures through the evaluation of the effect of different liposome preparation parameters, such as the dilution factor, sonication, chemical formulation and purification, on the DPPC:FA liposome characteristics. In a later stage, in-depth kinetic studies were carried out, aiming to provide good knowledge on the impact of the liposome encapsulation in the hydrogel matrix and on the effect of a magnetic field on the FA release kinetics and mechanisms while determining the possible use of these magnetic liposomal delivery systems in long-term treatments.

## 2. Materials and Methods

### 2.1. Materials

Gelatin from porcine skin, potassium phosphate monobasic (≥99%), sodium phosphate dibasic (≥99%), sodium chloride (≥99%), potassium chloride (≥99%), chloroform (>99%), methanol (>99%), glycerol (>99.5%) and Trans-Ferulic acid were all purchased from Sigma-Aldrich (St. Louis, MO, USA). 16:0 PC (DPPC) 1,2-dipalmitoyl-sn-glycero-3-phosphocoline was obtained from Avanti Polar Lipids, Inc. (Alabaster, AL, USA).

### 2.2. Methods

#### 2.2.1. Synthesis and Characterization of the Iron Oxide Nanoparticles

MNPs were prepared following the experimental procedures described in a previous publication [58]. Briefly, the MNPs were synthesized, in alkaline media, by chemical co-precipitation of two iron salts: FeCl_3_ and FeCl_2_. An aqueous solution of 25% (*v*/*v*) ammonium hydroxide was added to the salt mixture, under permanent stirring at 1250 rpm, at 80 °C, in a N_2_ atmosphere. The MNPs synthesized were characterized by transmission electronic microscopy (TEM) using a Hitachi H8100 TEM with a LAB6 filament and an acceleration tension of 200 kV to access the MNPs’ size and size distribution.

#### 2.2.2. Liposome Preparation, Loading and Purification

Multilamellar vesicles (MLVs) composed of 1,2-dipalmitoyl-sn-glycero-3-phosphocholine (DPPC) were prepared using the thin-film hydration method following the procedures described by Pires et al. [59]. Briefly, it consisted of the dissolution of the lipidic component (DPPC) in an organic phase, which, in this case, was a methanol/chloroform (1:4) solution, followed by the formation of a lipidic film in the recipient walls by solvent evaporation by exposure to an inert gas stream (N_2_ gas stream) for three hours. Finally, the lipidic film was hydrated with a phosphate buffer solution (PBS) at 55 °C for at least 1 h under constant stirring at 320 rpms, leading to the formation of unloaded DPPC vesicle systems. The DPPC vesicles were exposed to different sonication times varying from 15 min to 45 min, aiming to avoid the presence of larger vesicle aggregates. These unloaded vesicle systems (DPPC-unloaded liposomes) were used as the control samples for comparative terms.

DPPC liposomes loaded with FA (DPPC:FA liposomes) were prepared following the methodology described above but including the FA loading step, which was carried out by adding the FA to the organic phase. The obtained vesicle systems were sonicated for different times varying between 15 min and 60 min. The effect of sonication time on liposome structural characteristics was determined based on dynamic light scattering (DLS) measurements, which allowed for the selection of the ideal sonication time based on the liposome size heterogeneity assessed by analysis of the polydispersity index (PI). 

Liposomes with different chemical formulations were prepared using a constant DPPC concentration of 2 mM and different FA concentrations in a way to obtain liposomes with 3:1, 10:1 and 30:1 relative DPPC:FA mass fractions in order to evaluate the influence of chemical formulation on the liposome dimension, size heterogeneity, encapsulation efficiency (% EE) and loading capacity (% LC).

Liposomes were purified using a dialysis bag of regenerated cellulose with a molecular weight cut-off of 14 kDa (Spectra/Pro, Biotech, USA) for removal of the unloaded FA remaining in solution after liposome formation. The dialysis bag was filled with the liposomal solution and immersed in a dialysate PBS solution, at pH 7.4, with a volume 100× higher than that of the liposome solution, under constant stirring for 40 h, at room temperature. The dialysate was exchanged after 18 h. The dialysates collected at 18 h and 40 h were analyzed by UV-Vis, and the absorbance was collected in a range from 200 nm to 900 nm to verify the efficiency and completeness of the unloaded FA removal.

#### 2.2.3. Characterization of the Liposomes

##### Determination of Liposome Dimension

The dimensions of the loaded (DPPC:FA) and unloaded (DPPC) liposomes over time were determined through dynamic light scattering (DLS) using a nanoparticle analyzer (Nano Partica SZ-100, Horiba Scientific (Kyoto, Japan)). DLS measurements were performed in triplicate after liposome synthesis and purification, allowing for the determination of liposome size distribution, the mean size, the polydispersity index (PI) and the respective standard deviations.

##### Entrapment Efficiency (EE) and Loading Capacity (LC) of Ferulic Acid-Loaded Liposomes

The FA entrapment efficiency (EE) was determined for all the liposome suspensions according to Equation (1).

(1) $$EE\%=\frac{M_{Encap}}{M_{Total}}\times 100$$

where M_Encap_ is the amount of FA encapsulated in liposomes after dialysis (g) and M_Total_ is the initial amount of FA (g). The M_Encap_ was calculated by subtraction of the total amount of FA used to prepare the liposomal solution by the amount of FA released during dialysis. The amount of FA released during dialysis was assessed by determination of the absorbance at 311 nm (corresponding to the characteristic absorbance band of FA) in the dialysate samples collected over the dialysis time. The FA absorbance was converted to concentration using the Lambert–Beer equation and then to the absolute FA mass value by considering the dialysate volume used in liposome dialysis.

The entrapment efficiency (EE) of FA was confirmed by FA quantification upon liposome lysis. In this case, liposomes loaded with FA were added, after dialysis, to a 10% methanol solution to induce the release of FA upon liposome destruction. The released FA was quantified by determination of the absorbance at 311 nm as described above.

The drug loading capacity (LC) was also calculated using Equation (2).

(2) $$LC\%=\frac{M_{Encap}}{M_{Lipid}}\times 100$$

where M_Encap_ is the same parameter as in the previous equation and M_Lipid_ is the total amount of lipid used to prepare the liposomal formulation.

#### 2.2.4. Preparation of the Liposomal Hydrogel Membranes

The DPPC:FA liposomes were encapsulated into gelatin matrices/membranes by using active and passive approaches immediately after dialysis to minimize the possibility of liposomal aggregation and fusion. Active encapsulation was carried out by diffusion of unloaded (DPPC liposomes) and FA-loaded DPPC liposomes (DPPC:FA liposomes) into magnetic-responsive gelatin membranes previously prepared according to the procedure described by Manjua et al. [58]. Briefly, gelatin matrices were prepared by flat casting an 8% (*w*/*v*) porcine skin gelatin solution, containing 0.25% (*w*/*v*) of dispersed MNPs, in a silicone round mold and keeping it at 4 °C overnight for gelification. The gelatin hydrogel membranes doped with the MNPs were gently removed from the silicone round mold after gelification and crosslinked by immersion in 50 mL of an aqueous solution containing 1% (*v*/*v*) of glutaraldehyde (GA) for three hours at 4 °C. After crosslinking, the hydrogel membranes were washed with Milli-Q water for the removal of loosely bound components. Membrane washing was performed by immersion of the membranes in 10 mL of Milli-Q water under permanent stirring at 220 rpm. The washing solution was renewed every 10 min and analyzed in a UV-Vis spectrophotometer in a range from 200 nm to 900 nm. This washing procedure was repeated until the total disappearance of the bands corresponding to the release of unbound materials (loosely bound GA, gelatin molecules and MNPs). A minimum of five washing cycles were needed for complete membrane washing.

Passive encapsulation of the liposomes was performed by direct mixing of the liposomal solution into 8% gelatin solution at 40 °C. This mixture was stirred at 300 rpms for 15 min. This gelatin solution was then cast on a silicone round mold (0.2 mL/cm^2^) and kept at 4 °C, overnight, for gelification.

#### 2.2.5. Characterization of the Hydrogel Membranes

##### Swelling Ability

Small hydrogel membrane pieces of 1 cm^2^ were cut and dried for 2 h in a fume hood. The membrane samples were immersed in 5 mL of PBS buffer (pH 7.4) and kept in an incubator at 37 °C under constant stirring at 220 rpm. The membrane weight and dimensions were determined before the immersion and frequently monitored during membrane immersion for 28 h. The swelling ratio was calculated using Equation (3).

(3) $$\%Swelling\;ratio=\frac{W_{S}-W_{d}}{W_{d}}\times 100$$

where W_s_ and W_d_ correspond to the weight of swollen and dry hydrogel membranes, respectively.

##### Determination of the Hydrophilic Character and Water Uptake

The surface properties of the hydrogel membranes were assessed by determining the surface contact angle using a drop shape analyzer (KSV, CAM 100), coupled to a video camera for image acquisition. The surface contact angles were determined using glycerol as a solvent, instead of water, to minimize the penetration of the drop into the hydrogel matrix. The contact angles were determined as the average of 6 measurements.

##### Analysis of the Structural Integrity of the Liposomes-Encapsulated Hydrogels

The liposomal-based hydrogels were analyzed by optical microscopy to infer the impact of the encapsulation process on the structural integrity of the liposomes. Optical microscopy analyses were performed using an optical microscope (Nikon, Eclipse Ci) adapted to a digital camera (ProgRes CT3). The liposomal-based hydrogel membranes were analyzed with 10× eyepiece magnification and objective magnification ranging from 4× to 50×.

##### Chemical Characterization of the Liposomal Hydrogels

ATR-FTIR spectroscopy analysis was performed to evaluate the hydrogel crosslinking and for inspecting the presence of possible interactions of MNPs and DPPC liposomes with the gelatin matrix. ATR-FTIR spectra were collected using an FT-IR spectrometer (PerkinElmer (Shelton, CT, USA), Spectrum Two) in the spectral region from 400 to 4000 cm^−1^, at a 1 cm^−1^ resolution.

#### 2.2.6. Ferulic Acid Release Assay

FA release assays were performed, aiming at characterizing the FA release kinetics and mechanisms from liposomal hydrogel membranes while understanding the contribution of the liposome, hydrogel network and magnetic field in the regulation of the FA release. The role of the liposome and magnetic field stimuli on the controlled release of FA was determined by a comparative analysis of the FA release from liposomal hydrogels with that obtained from hydrogels doped with FA (without liposomes) and from DPPC:FA liposomal suspensions. The FA release was conducted in the presence and absence of a magnetic field to assess the effect of magnetic stimulation on the FA release profiles. 

The liposomal hydrogel systems used in the release assays were prepared by passive encapsulation of 3:1 and 10:1 DPPC:FA liposomes in an 8% (*w*/*v*) porcine gelatin solution containing dispersed MNPs with concentrations of 0%, 0.25% and 1% (*w*/*v*) and crosslinked with GA, as described in Section 2.2.4.

In this regard, small pieces of the different hydrogel membranes of 1 cm^2^ were immersed in 5 mL of fresh PBS and placed in an incubator with an orbital shaker (Incubating Light Duty Orbital Shakers, Ohaus, Parsippany, NJ, USA) at a controlled temperature of 37 °C (physiological temperature). The PBS solution was frequently sampled over time and analyzed in a UV-Vis spectrophotometer for absorbance spectra acquisition at the spectral region of the FA, considering the absorbance band at 311 nm to avoid superimposition with the absorbance signal from proteins at 280 nm and possible interferences from dissolved gelatin. In the experiments conducted with magnetic field stimulation, a neodymium bar allowing a magnetic field intensity of 0.08 T was placed under the beaker containing the immersed hydrogel sample.

The release assays from liposomal solutions were carried out following identical procedures. However, in this case, the liposome solution was kept inside a regenerated cellulose dialysis bag with a molecular weight cut-off of 14 kDa (Spectra/Pro, Biotech, USA), which was then immersed in a PBS dialysate solution (pH 7.4) and placed inside an incubator with controlled temperature at 37 °C under constant orbital shaking. The FA release was monitored by periodic measurement of the dialysate absorbance at 311 nm. In this case, the resistance of the dialysis bag to the FA transport was considered negligible, considering the differences between the MWCO of the dialysis bag (14 kDa) and the FA molecular size (194.18 g/mol). 

The FA release kinetics and mechanisms were studied by adjustment of the experimental data points to release kinetic models, such as zero-order, 1st-order, Higuchi and Korsmeyer–Peppas models expressed by Equations (4)–(7) and their respective linearized forms [60,61].

(4) $$\frac{dM_{R}}{dt}=k,\;\mathrm{zero-order}\mathrm{model}$$

$$M_{R}=kt,\;\mathrm{linearized zero-order model}$$

(5) $$\frac{dM_{R}}{dt}=k{\;\times \;M}_{R},\;\mathrm{1st-order model}$$

$$Ln\left(M_{R}\right)=Ln(M_{R0})\times kt,\;\mathrm{linearized 1st-order model}$$

(6) $$M_{R}=k_{H}t^{0.5},\;\mathrm{Higuchi model}$$

$$Log\left(M_{R}\right)=Log\left(k_{H}\right)+0.5Log(t),\;\mathrm{linearized Higuchi model}$$

(7) $$\frac{M_{R}}{M_{T}}=k_{KP}\times t^{n},\;\mathrm{Korsmeyer–Peppas model}$$

$$Log\left(\frac{M_{R}}{M_{T}}\right)=Log\left(k_{KP}\right)+nLog(t),\;\mathrm{linearized Korsmeyer–Peppas model}$$

where M_R_ is the amount of the FA released to dissolution media at time t, M_T_ is the total amount of FA in the delivery platform (i.e., the hydrogel membrane, the liposome and the liposomal hydrogel) at the beginning of the experiment (t = 0 min), n is the exponential diffusion, and k_0_, k_1_, k_H_ and k_KP_ are the zero-order, 1st-order, Higuchi and Korsmeyer–Peppas release constants, corresponding to the constant release rates.

The FA release kinetics and mechanism were determined by a comparative analysis of the values obtained for the different release parameters.

## 3. Results and Discussion

### 3.1. Synthesis and Characterization of FA-Loaded Liposomes

The first stage of this work was focused on the synthesis of liposomes with fine-tuned characteristics, i.e., low-size heterogeneity, aiming to obtain monodispersed liposomal suspensions and thus avoiding the presence of vesicle aggregates. In this regard, the effect of different liposome preparation variables, such as the dilution factor of the liposome suspensions, sonication time, chemical formulation and purification, on liposome dimension and size heterogeneity was studied to ensure the optimal adjustment of the parameters, which may lead to the preparation of liposomes with desirable characteristics. The liposome dimension and size heterogeneity were evaluated based on the vesicle mean size and the polydispersity index (PI) values obtained by DLS analysis. 

Table 1 shows the mean size and PI values obtained for unloaded liposomes with different dilution factors and prepared by exposure to different sonication times.

**Figure A1:**
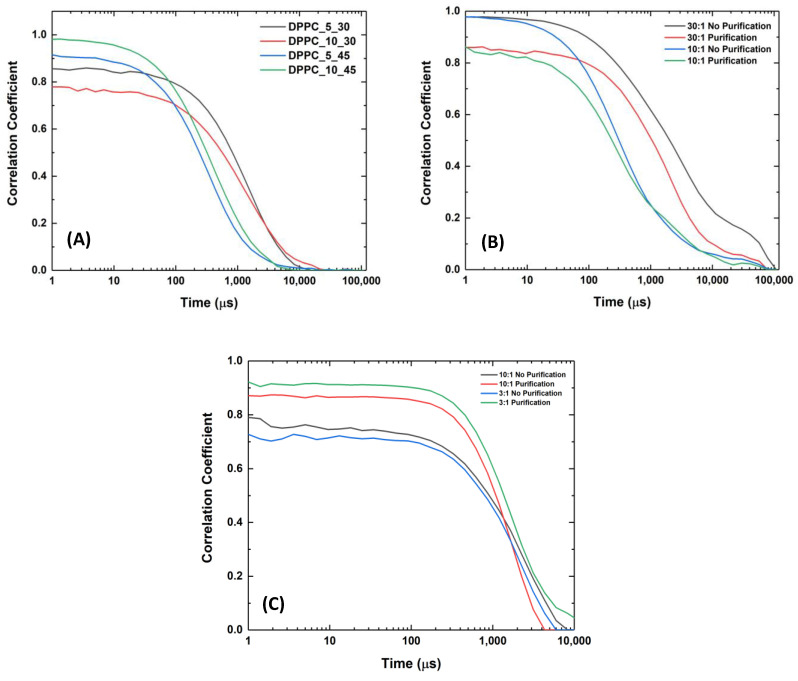
DLS correlograms obtained for (**A**) empty DPPC liposomes dispersed in PBS solution with different dilution factors (5× and 10×) and exposed to different sonication times (30 and 45 min); (**B**) 30:1 and 10:1 DPPC:FA liposomes, upon 45 min of sonication, before and after purification by dialysis; and (**C**) 3:1 and 10:1 DPPC:FA liposomes upon 60 min of sonication, before and after purification by dialysis.

As shown in Table 1, a remarkable decrease of ca. 60% in the liposome mean sizes was observed with the increase in the sonication time from 30 min to 45 min. The observed decrease in liposome sizes from 500–700 nm to the 200–300 nm range suggested an increase in the content of small unilamellar vesicles (SUVs) to the detriment of the multilamellar vesicles (MLVs). The increase in the dilution factor also led to a decrease in the liposomal size, but, in this case, the effect was much less expressive than that obtained by the increase in the sonication time. The effect of the sonication time and the dilution factor on the PI values was not totally evident. However, PI values varying from 0.51 ± 0.12 and 0.53 ± 0.09 were consistently obtained when increasing the sonication time to 45 min. The DLS correlograms respective to the liposomes described in Table 1 were added in Figure A1A in Appendix A for a better perception of the effect of the sonication time on the liposome polydispersity (PI). A comparative analysis of the DLS correlograms in Figure A1A shows that the increase in the sonication time is followed by a narrowing of the correlation function, compatible with the increase in liposome size homogeneity (e.g., higher size monodisperse character) in suspension.

The impact of FA loading in the liposome and the liposome purification step on the liposome morphology was then inspected. DPPC liposomes loaded with FA (DPPC:FA liposomes) were prepared using different DPPC:FA ratios of 3:1, 10:1 and 30:1. Table 2 shows the mean size and PI values obtained for a non-diluted solution of the DPPC:FA liposomes, upon 60 min sonication, before and after purification by dialysis.

The sonication time was further increased from 45 min (Table 1) to 60 min (Table 2), allowing for an additional reduction in the liposome size to 106.6 ± 45.9 and size polydispersity, expressed by a reduction in PI values of the unloaded DPPC, to 0.36 ± 0.34. The impact of sonication time on the dimension of FA-loaded liposomes was also studied (Table A1 in Appendix A), and it was observed to produce a significant decrease in the PI values from the value of 0.63 ± 0.05 obtained for DPPC:FA 10:1 liposomes (Table A1 in Appendix A) to 0.12 ± 0.01 for DPPC:FA 10:1 liposomes (Table 2). No significant changes were noticed in DPPC:FA liposomes with a lower FA fraction, i.e., DPPC:FA 30:1 liposomes, suggesting a possible role of FA in the structural characteristics of these liposomes. In fact, Table 2 also shows that the encapsulation of FA led to an increase in the liposomal size, more evident in liposomes with higher FA fractions, i.e., 3:1 and 10:1 DPPC:FA liposomes, followed by a significant decrease in the PI to half of its value, thus evidencing the positive contribution of FA for the size homogeneity of the liposomal suspension.

**Figure 1:**
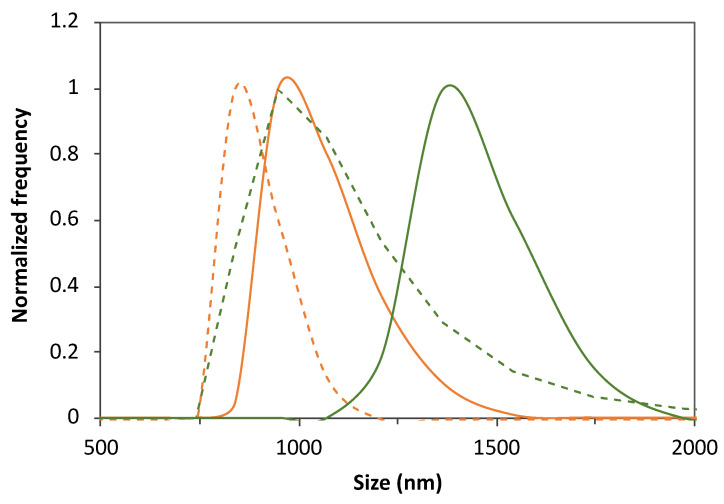
Illustration of the effect of FA loading, purification by dialysis and DPPC:FA formulation on the liposome size distribution. Size distribution of 3:1 (orange lines) and 10:1 (green lines) DPPC:FA liposomes before (solid lines) and after (dashed lines) dialysis.

Also, liposome purification by dialysis was found to have a remarkable effect on the liposome size, leading to a significant decrease in the average size of the liposomes from the 1200–1400 nm range to values below the microscale dimensions (<1000 nm), as illustrated in Figure 1 and Table 2. Minor changes were observed in the mean size of liposomes formed by the lowest FA proportion of 30:1 DPPC:FA. The liposome mean sizes obtained before purification suggested the predominance of MLVs with the potential presence of vesicle aggregates. Yet, despite the decrease in the average liposome sizes, MLVs were still predominant after dialysis, with the 3:1 DPPC:FA sizes varying from 750 nm to 1000 nm, whereas the 10:1 DPPC:FA sizes were mostly contained in the 750 nm–1750 nm range, as illustrated in Figure 1. The effect of dialysis was also noticed in the DLS correlograms of purified and non-purified liposomes shown in Figure A1B, confirming the efficiency of dialysis in removing possible forming aggregates and large vesicles present in suspension. However, it is noteworthy that these changes in the liposome size distribution did not have a pronounced effect on the size heterogeneity of liposomes with higher FA content (3:1 and 10:1 DPPC:FA liposomes), as reflected by the insignificant changes in PI values (Table 2). Figure A1C also illustrates the DLS correlograms obtained for DPPC:FA liposomes after dialysis, prepared by an optimized selection of the liposome synthesis variables considered in the present work. 

The entrapment efficiency (EE) and loading capacity (LC) obtained for FA-loaded DPPC liposomes prepared using different DPPC:FA ratios were determined using Equations (1) and (2), respectively. The results obtained are listed in Table 3.

The results depicted in Table 3 show that the increase in the FA fraction in the liposomal formulations led to the increase in entrapment efficiency, EE, and loading capacity, LC. This result was already expectable as it results from the higher availability of FA for encapsulation in the lipidic bilayer of liposomes per amount of the lipidic component.

Considering the characteristics of the prepared DPPC:FA liposomes described above, the MLVs with the highest fractions of FA, i.e., 10:1 and 3:1 DPPC:FA, were selected for the development of the liposomal hydrogels described in the following sections of this paper.

### 3.2. Design and Characterization of the Liposomal Gelatin Membranes

The main goal of this work was to develop liposomal hydrogel delivery systems allowing for a magnetically modulated and long-term drug release while providing a deeper understanding of the impact of liposomal encapsulation and the effect of the magnetic field on the release mechanisms and kinetics of small bioactive molecules with poor bioavailability. In this regard, FA, a small organic bioactive molecule with anti-inflammatory and pro-angiogenic properties, was used as a representative model molecule in this work.

The magnetic-responsive liposomal hydrogels were prepared by encapsulation of the MLV DPPC liposomes loaded with FA (3:1 and 10:1), previously described and listed in Table 2, into gelatin membranes containing dispersed iron oxide nanoparticles (MNPs)—magnetic-responsive gelatin. On the one hand, the liposomes were used as drug reservoirs offering a more efficient imprisonment of small therapeutic molecules than that achieved by direct drug immobilization in hydrogel matrices. On the other hand, the unique mechanical behavior of magnetic-responsive hydrogels (magnetic-responsive gelatin in the case of the present study) in the presence of magnetic stimuli was explored for improved in situ and on-demand regulation of FA delivery. 

#### 3.2.1. Encapsulation of Liposomes into the Gelatin Membranes

MLVs are vesicles consisting of multiple concentric lipid bilayers, which are considered less effective drug delivery systems than SUVs as they are more prone to fusion and aggregation, which may result in the loss of their enclosed payload. Furthermore, the possible immunogenicity and toxicity of MLV liposomes can also present difficulties; hence, SUVs tend to be preferred for more accurate targeting and effective cellular uptake. However, MLVs present specific characteristics that make them more attractive considering the primary objective of the present work, which is the development of liposomal hydrogel systems allowing for long-term drug storage and transportation. MLVs are larger than SUVs, allowing for a higher capacity for drug loading, and their multilayered lipid bilayers enable higher tolerance to harsh conditions such as pH changes or temperature variations while working as additional barriers to drug transport, delaying drug release and thus providing a controlled and prolonged drug release profile. Furthermore, the additional premises of using MLVs were that (i) MLVs largely remain intact during the hydrogel preparation process (crosslinking method), contrary to SUVs (higher tendency to rupture); (ii) the mechanical stability of SUVs, even in a hydrogel matrix, can be lower compared to the MLVs; and (iii) the lower size of SUVs can enable their easier diffusion through the gelatin hydrogel, and their unilamellarity tends to destabilize and immediately releases high levels of ferulic acid (burst release).

**Figure 2:**
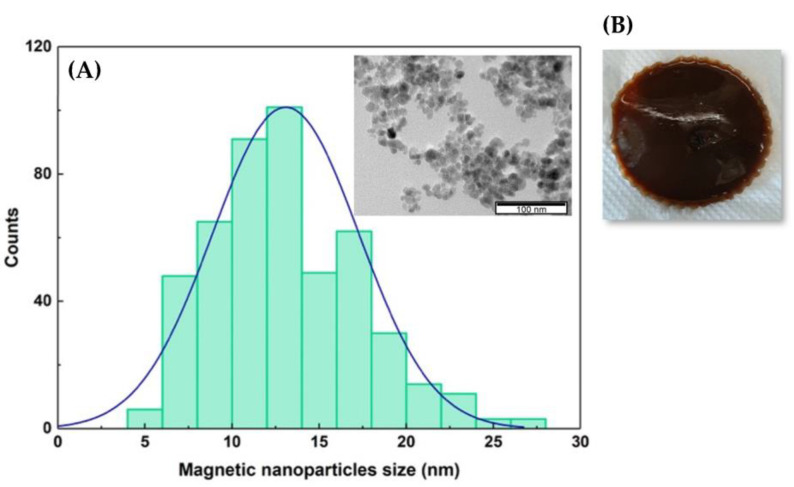
(**A**) Size distribution of the MNPs adjusted to a Gaussian function. Inset. Image of the MNPs obtained by transmission electron microscopy (TEM). (**B**) Photo of the magnetic-responsive gelatin doped with 1% of MNPs.

The encapsulation of DPPC:FA liposomes into gelatin membranes was attempted by active and passive immobilization procedures. Active immobilization involved the diffusion of the liposomes from the liposomal solution by swelling of the pre-formed magnetic-responsive gelatin membranes crosslinked with glutaraldehyde. Passive immobilization consisted of the direct addition of the liposome solution to the gelatin solution containing dispersed MNPs with an average size of 12.7 nm (Figure 2A) before casting and gelification. The liposomal gelatin membranes were then immersed in a glutaraldehyde solution for crosslinking, rendering magnetic-responsive gelatin membranes resembling the one shown in Figure 2B.

**Figure 3:**
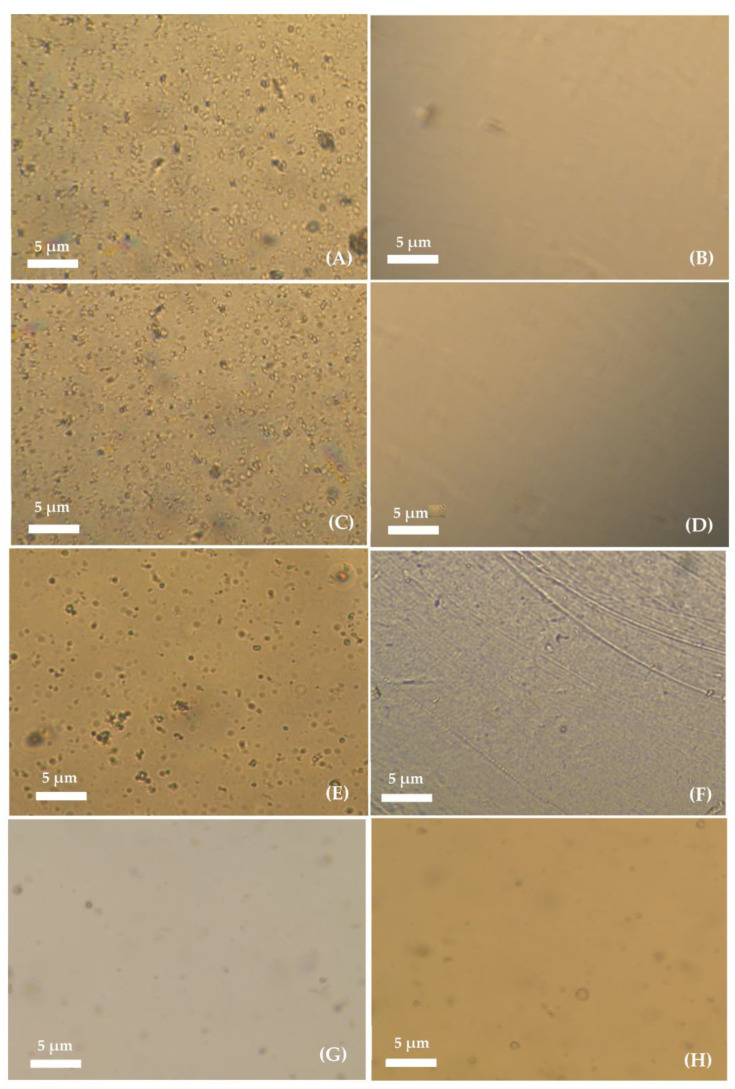
Images of the non-crosslinked liposomal-based hydrogel prepared by active immobilization of DPPC:FA liposomes acquired (**A**) before and (**B**) after washing for 6 h; liposomal-based hydrogel prepared by active immobilization of DPPC:FA liposomes in a gelatin membrane crosslinked for 3 h acquired (**C**) before and (**D**) after washing for 6 h; liposomal-based hydrogel prepared by active immobilization of DPPC:FA liposomes in gelatin membranes crosslinked for 6 h acquired (**E**) before (**F**) and after washing for 6 h; and liposomal-based hydrogel prepared by passive immobilization of DPPC:FA liposomes crosslinked for 3 h acquired (**G**) before and after (**H**) washing for 6 h. Hydrogel washing was performed through immersion of the hydrogels in PBS solution with pH 7.4 at 37 °C. All images were taken with 10× magnification with 10× eyepiece.

The liposomal gelatin membranes obtained by active and passive liposome encapsulation were inspected by using optical microscopy. Microscopy analysis of the liposomal hydrogels was performed for evaluation of the efficiency of the different experimental approaches used for encapsulation based on the presence of the encapsulated liposomes, the liposome dispersion quality and structural integrity. Figure 3 shows the images obtained through optical microscopy of the liposome hydrogel membranes with different crosslinking degrees, prepared by active and passive immobilization of DPPC:FA liposomes, before washing (on the left). The images shown on the right side correspond to the same respective gelatin membranes after washing by immersion for different durations (from 0 to 6 h) in PBS at pH 7.4 and 37 °C. 

Several randomly dispersed vesicles resembling the typical structure of liposomes and/or liposome aggregates were identified before hydrogel washing in hydrogels prepared by active immobilization. A comparative analysis of Figure 3A,C,E seems to indicate that the number of immobilized vesicles depended on the hydrogel crosslinking degree, decreasing with the increase in the hydrogel crosslinking reaction time from 3 h to 6 h. The decrease in the number of immobilized vesicles was possibly explained by the limited diffusion of the liposomes into the hydrogel network, which was expected to increase with the increase in the crosslinking degree due to the reduction in the dimension of the hydrogel voids. An inspection of the microscopy images obtained for liposomal hydrogel membranes after washing, as shown in Figure 3B,D and F, revealed the total loss of the liposomal vesicles from the membranes during the washing process, suggesting that active immobilization led to a non-efficient internalization of liposomes into the gelatin matrix. 

The removal of liposomes from the hydrogel membranes was possibly due to the limited access of liposomes to hydrogel cavities with reduced dimensions or located in less accessible regions of the crosslinked hydrogel, which would assure more efficient imprisonment of the liposomes. The limited diffusion of liposomes into the hydrogel network forced their location at the surface of the hydrogel and in the larger cavities in the hydrogel matrix, allowing for easier liposome removal by back diffusion to the clean washing solution, upon hydrogel swelling at 37 °C, due to the osmotic difference effect. 

Finally, the passive immobilization of liposomes was tested, aiming at reducing the diffusional limited encapsulation of the liposomes. The microscopy images obtained for liposomal hydrogels prepared by passive immobilization in Figure 3G,H seem to indicate a much lower number of entrapped vesicles than that observed for liposomal hydrogels prepared by active immobilization, before washing (Figure 3G). Despite the reduced number, the immobilized vesicles showed good structural integrity and a liposome-like structure, and, thus, they were also ascribed to the presence of liposomes (non-discarding the possible presence of liposomal aggregates). 

The comparative analysis of the hydrogel images before (Figure 3G) and after washing (Figure 3H) revealed a minimal loss of liposomes during the washing step, evidencing that passive immobilization allowed for more efficient development of liposomal hydrogel delivery systems with improved stability (with respect to the capacity to retain the encapsulated liposomes).

The ability of the gelatin membranes to retain and stabilize the encapsulated liposomes and to regulate FA release is very much dependent on their swelling capacity. The swelling ability is influenced by the temperature, the capacity of the crosslinking agent to covalently bind two polymer chains and the potential contribution of the embedded liposomes. Liposomes might possibly produce two opposite effects: 1. liposomes may be able to interact with the gelatin molecules, increasing the structural cohesion of the hydrogel matrix and contributing positively to the hydrogel stability but negatively to the hydrogel swelling capacity, and 2. liposomes may potentially perturb the access of the crosslinking agent to the gelatin molecules through a stereochemical effect, thus reducing the crosslinking efficiency. In this last case, the liposome would exert a negative contribution in terms of hydrogel stability and a much possible positive contribution in terms of the hydrogel swelling capacity.

#### 3.2.2. Determination of the Liposomal Gelatin Membranes Swelling Ability

Studies were performed to evaluate the effect of liposomes and MNPs on the swelling ability of the gelatin membranes under specific physiological conditions, i.e., at pH 7.4 and 37 °C.

**Figure 4:**
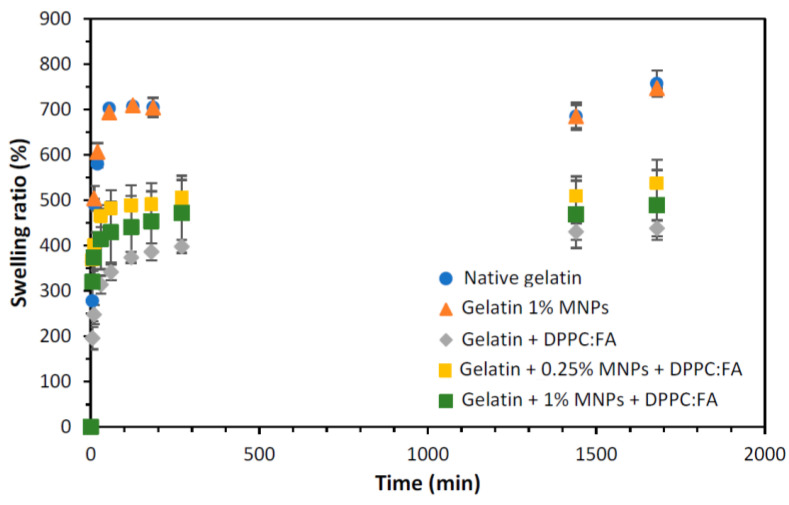
Swelling ratio obtained for gelatin membranes without (dark blue circles) and with 1% of MNPs (orange triangles), gelatin membranes with internalized DPPC:FA liposomes (grey diamonds) and magnetic-responsive gelatin membranes doped with 0.25% (yellow squares) and 1% MNPs (green squares) and with encapsulated 3:1 DPPC:FA liposomes, by immersion in PBS solution at 37 °C.

The swelling ratios of the gelatin membranes with embedded MNPs (magnetic-responsive gelatin membranes), gelatin membranes with encapsulated liposomes (without MNPs) and magnetic-responsive gelatin membranes containing 0.25% and 1% MNPs, with encapsulated liposomes, were determined using Equation (3) and compared with that obtained for native gelatin membranes, i.e., without any encapsulated component. The swelling ability of each liposomal gelatin membrane was determined based on an analysis of the swelling ratio profiles over time, represented in Figure 4.

The analysis of the swelling ratio profiles revealed a more accentuated increase in the gelatin membrane swelling during the first hour, reaching a plateau after 120 min. Native gelatin membranes and gelatin membranes with embedded MNPs showed identical swelling ratios, evidencing that the presence of MNPs (in the concentrations used in this study) does not significantly affect the swelling and water uptake capacity of these membranes. Contrastingly, a remarkable decrease in the maximum swelling was observed for gelatin membranes with immobilized DPPC:FA liposomes, suggesting the contribution of these liposomal structures to an additional cohesion of the gelatin matrix. A similar effect has been reported by other authors. Wu et al. [46] and Lee et al. [62] showed that the encapsulation of liposomes in hydrogel matrices improved the hydrogel strength, increasing the compressive modulus of the liposomal gel and thus justifying a higher resistance to deformation and lower swelling capacity. In a previous work, Lee et al. [62] explained the increase in the strength of gelatin-DPPC liposomal systems due to the interaction of liposomes with the hydrophobic moieties in a gelatin network, which acted as additional crosslinkers. However, given the diversity of the functional groups present in a protein chain and the high hydrophilicity of the hydrogel, the crosslinking effect of liposomes may also be plausibly explained by the interaction of the polar phospholipidic heads of the DPPC located at the external surface of liposomes with the hydrophilic moieties present in the gelatin chain. 

The active contribution of the liposomes in hydrogel crosslinking may explain the higher retention of liposomes in liposomal hydrogel systems prepared by passive immobilization, as the addition of liposomes to the polymer solution before gelification may render the imprisonment of liposomes in more internal hydrogel regions, followed by a decreased liposome back diffusion ascribed to the lower swelling ability of these liposomal gelatins.

#### 3.2.3. Chemical Characterization of the Liposomal Gelatin Membranes

**Figure 5:**
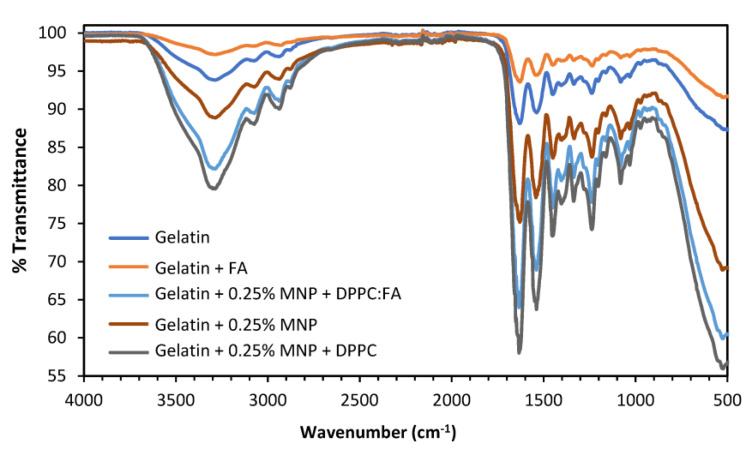
ATR-FTIR spectra for the native gelatin membranes with (orange line) and without (dark blue line) immobilized FA (without liposomes) and magnetic-responsive gelatin doped with 0.25% MNPs without liposomes (brown line), with unloaded liposomes (dark grey line) and with liposomes loaded with FA (light blue).

The ATR-FTIR spectra of the liposomal gelatin membranes were determined and analyzed to obtain information on the chemical characteristics of the gelatin membranes and to confirm the presence of FA, MNPs and DPPC:FA liposomes in magnetic-responsive liposomal gelatin membranes. Figure 5 shows the transmittance spectra obtained for the different gelatin membranes.

Similar FTIR spectra were obtained for all membranes analyzed, mostly characterized by the presence of the bands located in the same spectral region, indicating that the presence of FA, MNPs and DPPC:FA liposomes, possibly due to their small amounts in the membrane, have little influence on the FTIR spectra. A broad band identified between 3600 cm^−1^ and 3100 cm^−1^ was attributed to the -NH and -OH stretching vibrations [63], visible for all samples. The spectral region from 1700 cm^−1^ to 800 cm^−1^ shows major spectral bands at 1630 cm^−1^, 1539 cm^−1^ and 1239 cm^−1^, characteristic of gelatin spectra [63] and attributed, respectively, to the C=O and C-N stretching vibrations of the amide carbonyl group in Amide I, the N-H and C-N stretching vibrations of groups in Amide II and C-N and N-H stretching vibrations in Amide III.

A small spectral band at 523 cm^−1^ only observed for gelatin membranes embedded with MNPs was attributed to the presence of the MNPs. As reported in further literature, MNP’s characteristic band is located from 580 cm^−1^ to 400 cm^−1^ [64], denoting a spectral shift for MNPs embedded in the gelatin membranes. This spectral shift from 580 cm^−1^ to 523 cm^−1^ might be explained by the low MNP concentrations, which make their detection difficult. 

FA is the most difficult element to identify in these spectra, which may result from its low solubility in aqueous phases, justifying a low FA content outside liposome structures. Actually, the quantification of FA performed through UV-Vis analysis, after the destruction of a membrane containing 0.25% MNPs and embedded DPPC:FA liposomes, showed that the total amount of FA in the membranes was as low as 2.5 × 10^−5^ g/cm^2^. Furthermore, most of the characteristic spectra bands of FA are located between 1668 cm^−1^ and 685 cm^−1^ and are coincident with many of the characteristic spectral bands of gelatin, which also perturbs the detection of FA spectral signals. 

### 3.3. Ferulic Acid Release Assays

The FA release profiles obtained from liposomal gelatin membranes embedded with 3:1 and 10:1 DPPC:FA liposomes were determined and compared with those obtained with native gelatin membranes with immobilized FA and with a free MLVs DPPC:FA liposome suspension in order to understand the impact of the hydrogel matrix on FA release from liposome delivery systems.

The liposomal hydrogels used for FA release assays were prepared via passive encapsulation of DPPC:FA liposomes, as previously described, and all FA release experiments were conducted at pH 7.4 and 37 °C to mimetic the physiological conditions of the human organism.

**Figure 6:**
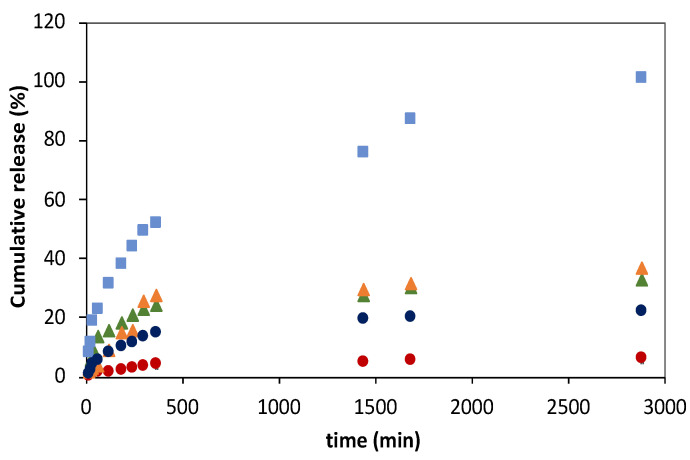
Ferulic acid (FA) release profiles from gelatin membranes with immobilized FA (light blue squares), from liposome suspensions prepared using 3:1 (green triangles) and 10:1 (orange triangles) DPPC:FA fractions and from magnetic−responsive liposomal gelatin membranes doped with 0.25% MNPs containing encapsulated 3:1 (red circles) and 10:1 (blue circles) DPPC:FA liposomes at 37 °C. The cumulative release of FA was determined by M_R_/M_T_ × 100.

As shown in Figure 6, the FA release profiles obtained for all delivery systems studied were characterized by a faster FA release in the initial process stage, followed by a decrease in the FA release rates over the process time, suggesting the dependence of the release rate on the FA concentration. This trend was, however, less notorious for liposomal gelatins with encapsulated 3:1 DPPC:FA liposomes, which showed a more linear and smoother FA release over time. The release of FA directly immobilized into the gelatin matrices was complete after 2880 min (48 h) in contrast to that observed for liposome suspensions and liposomal gelatin membranes, which showed delayed FA delivery, with maximum cumulative FA release reaching values of 36.8%, 32.9%, 22.1% and 6.2% for liposome suspensions and liposomal hydrogels with 10:1 and 3:1 DPPC:FA liposomes, respectively, over an identical experimental period of 48 h.

For a deeper comparative analysis, the FA release profiles were adjusted to different mathematical models, such as zero-order (Equation (4)) and first-order (Equation (5)) kinetic models and to the empirical Higuchi (Equation (6)) and Korsmeyer–Peppas (Equation (7)) models, aiming at obtaining a good understanding of the FA release mechanisms and kinetics from the different delivering systems.

**Figure A2:**
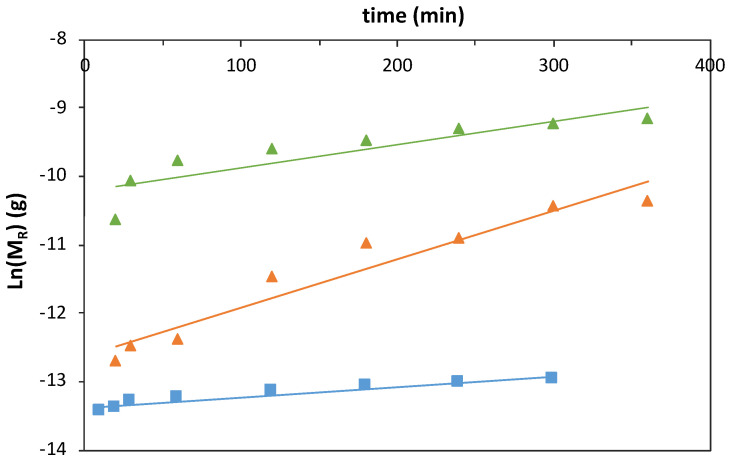
Logarithmic function of the ferulic acid (FA) mass release profiles obtained with magnetic−responsive gelatin membranes with embedded FA (blue squares) and liposome suspension with 10:1 (orange triangles) and 3:1 (green triangles) DPPC:FA fractions, at 37 °C, fit to 1st-order kinetic functions.

**Figure A3:**
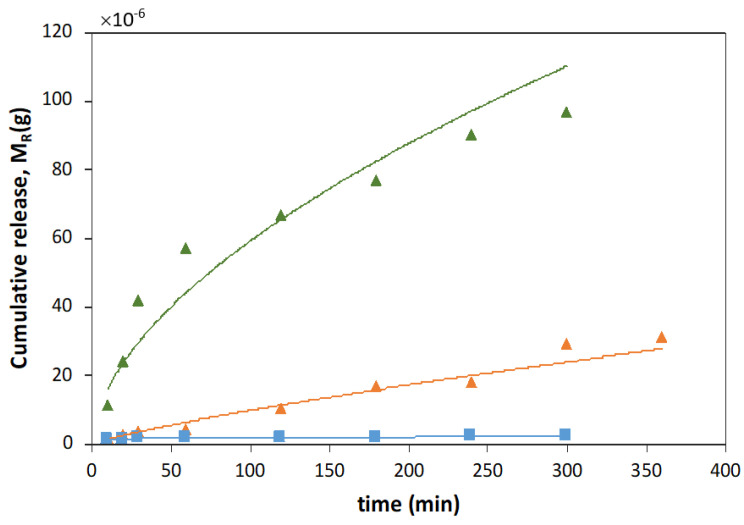
Ferulic acid (FA) mass release profiles obtained with magnetic−responsive gelatin membranes with embedded FA (blue squares) and liposome suspension with 10:1 (orange triangles) and 3:1 (green triangles) DPPC:FA fractions, at 37 °C, fit to Higuchi model.

The FA release obtained for the different delivery systems was found not to be properly described by zero-order kinetic models, since the cumulative FA release does not depend linearly on time. Also, FA release profiles were not best described by a 1st-order kinetic function (Figure A2, in Appendix A), as evidenced by R^2^ values < 0.94 (Table A2, in Appendix A), but they finely fitted to Higuchi models (Figure A3, in Appendix A), as expressed by R^2^ values of 0.968 and 0.983 (Table A3, in Appendix A). However, despite the good fitting quality, it is important to notice that diffusional exponent values of 0.641, 0.807 and 0.141 were obtained for native hydrogels with immobilized FA and for 3:1 and 10:1 DPPC:FA liposome suspensions, which are significantly different from the exponential diffusion value of 0.5, characteristic of a Higuchian release. For this reason, it is not possible to conclude that the FA release mechanisms in these delivery systems followed a Higuchian release mechanism.

**Figure A4:**
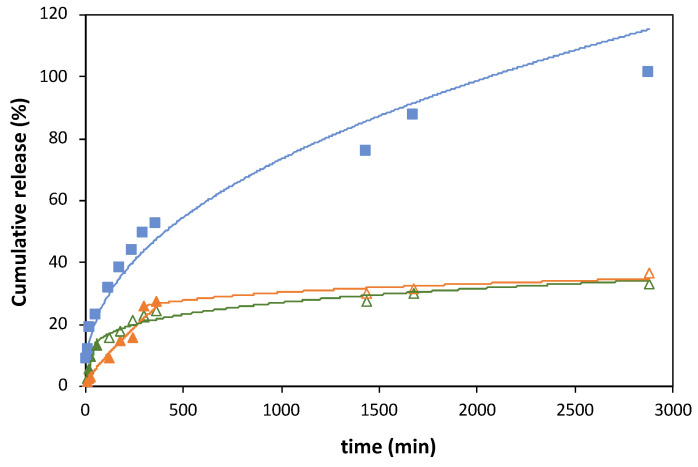
Ferulic acid (FA) release profiles obtained with magnetic−responsive gelatin membranes with embedded FA (blue squares) and liposome suspensions with 10:1 (orange triangles) and 3:1 (green triangles) DPPC:FA fractions, at 37 °C, fit to Korsmeyer–Peppas model. The cumulative release of FA was determined by M_R_/M_T_ × 100.

FA release profiles from gelatin membranes with immobilized FA and gelatin membranes with encapsulated 3:1 and 10:1 DPPC:FA liposomes were acceptably described by Korsmeyer–Peppas models as shown in Figure A4 in Appendix A, showing R^2^ values of 0.980, 0.934 and 0.915 (Table 4). 

**Figure 7:**
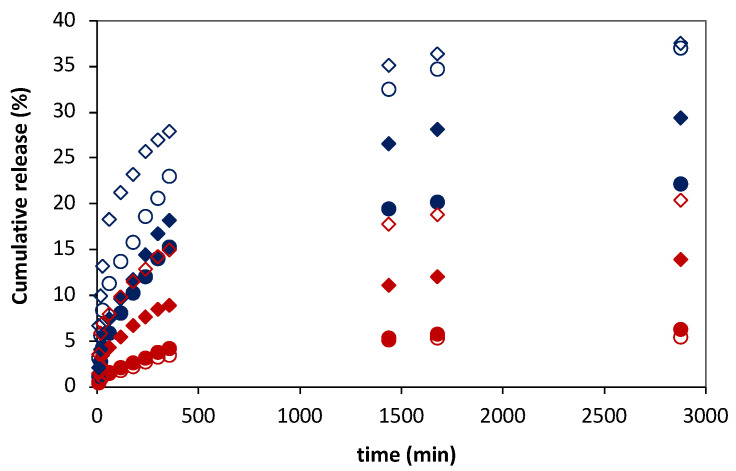
Ferulic acid (FA) release profiles from 3:1 (red symbols) and 10:1 (blue symbols) DPPC:FA liposomes embedded in magnetic-responsive gelatin membranes containing 0.25% MNPs (filled symbols) and 1% MNPs (empty symbols) in the absence (circles) and presence (diamonds) of magnetic field (MF) with an intensity of 0.08 T at 37 °C. The cumulative release of FA was determined by M_R_/M_T_ × 100.

The FA release mechanism of each delivery system was determined by analysis of the releasing parameters, i.e., the release constant k_KP_ and the diffusion exponent, n, obtained by adjustment of the linearized Korsmeyer–Peppas models to the experimental data points as shown in Figure 7.

The values obtained for the releasing parameters for each delivery system are listed in Table 4. The release constant, k, expresses the molecular releasing rates, whereas the diffusion exponent, n, indicates the transport mechanism underlying the FA release in each delivery system [60].

The diffusion exponent n was found to be closer to 0.5 for FA delivery from gelatin membranes with dispersed FA and from the DPPC:FA liposomes encapsulated within gelatin membranes, which indicates that the FA release in these systems occurs in a diffusional controlled regime [60], similarly to that previously reported [35]. A comparative analysis of the release constant k_KP_ revealed that the FA release process is significantly faster in gelatin membranes with dispersed FA, showing a release constant k_KP_ of 3.818, than that observed from DPPC:FA liposomes immobilized in gelatin membranes, characterized by k_KP_ values of 0.264 and 1.104 for liposomal hydrogel membranes containing 3:1 and 10:1 DPPC:FA liposomes, respectively, resulting in a remarkably delayed FA release, reaching maximum releasing values of ca. 20% in the same period of 48 h, as shown in Figure 6. The decrease in the FA release rates observed for liposomal hydrogels agrees with previous reports from other authors [35,38], being attributable to the imprisonment of FA into the liposomal vesicles, the higher resistance offered by liposomal hydrogels and the FA transport to the external media. Furthermore, it evidences the important regulatory effect of the liposomal structure in the FA release and its higher capacity to retain FA in comparison to that achieved with native gelatin membranes.

The results also unveiled that FA release from liposomal gelatin membranes is also dependent on the chemical formulation of DPPC:FA liposomes. DPPC:FA liposomes formed by a lower FA proportion, i.e., 10:1 DPPC:FA liposomes, allowed for a faster FA release than that observed for 3:1 DPPC:FA liposomes. Liposomal hydrogels containing dispersed 10:1 DPPC:FA liposomes allowed for 22.1% release of FA in 48 h, leading to a k_KP_ value of 1.104, which contrasts with the 6.2% FA release obtained with the liposomal hydrogels doped with 3:1 DPPC:FA liposomes in the same experimental period, which resulted in a k_KP_ value of 0.264. These results seem to be partially contradictory to those observed by Mourtas et al. [36]. These authors identically reported the existence of a dependence of the release rates of lipophilic molecules, in liposomal hydrogels, on the liposome payloads. However, in contrast with that observed in this work, they concluded that it increased with the increase in the drug load. The delayed FA release observed in the present work from liposomal hydrogels containing liposomes with higher FA loads (3:1 DPPC:FA liposomes) may hypothetically be explained by the higher stability of these liposomes due to a strong interaction between the FA and the aliphatic chains of the phospholipids in the lipidic bilayer. Due to its hydrophobic character, FA is more likely allocated within the lipidic bilayer than in the hydrophilic liposome core. FA will possibly act as a stabilizer of the lipidic bilayer, creating a more cohesive vesicle and thus enhancing the liposome stability with a consequent decrease in FA release. However, changes in the FA release profiles associated with differences in the structural morphology and mechanical behavior of these liposomal gelatins caused by the different dimensions of the encapsulated liposomes cannot be excluded.

The FA release mechanisms obtained for the hydrogel-based delivery systems contrast with those obtained with DPPC:FA liposome suspensions. The FA-releasing profiles obtained for DPPC:FA liposome suspensions showed FA cumulative releases of 32.9% and 36.8% after 48 h for 3:1 and 10:1 DPPC:FA liposomes, respectively, corresponding to a delayed release of FA comparatively to that obtained with hydrogels with immobilized FA (Figure 6). These results reveal the higher ability of liposomes to regulate the release of small molecules than that obtained with the native gelatin hydrogels. The poorer drug delivery control of gelatin hydrogel membranes is attributed to the large dimension of the gelatin voids, characteristic of the swollen hydrogel networks, allied in this case to a low interaction of FA with the gelatin matrix, considering the different polarities of these two molecules. 

It is interesting to note that the analysis of the FA release profiles with the Korsmeyer–Peppas model seemed to indicate that the release of FA from liposome suspensions obeys a dual-release regime (Figure A4 in Appendix A). As observed in this figure, FA delivery consists of two different releasing stages, with each one described by a different Korsmeyer–Peppas function (Table 4), evidencing a change in the FA release mechanism at the mid-term process. As shown in Table 4, in the initial stage, the FA release was characterized by n values of 0.794 and 0.920, respectively, for 10:1 and 3:1 DPPC:FA liposomes, indicating that the FA release occurs through an anomalous Fickian diffusion. The second stage was characterized by an abrupt decrease in the exponential diffusion n to values < 0.3, followed by a strong increase in the release rate constant, k_KP_, from values lower than 0.5 in the first stage to values of 5.81 and 12.46 in the second process stage for 3:1 and 10:1 DPPC:FA liposomes, respectively. This change in the diffusion exponential value, n, evidences a change in the FA release mechanism from an anomalous Fickian diffusion, in the first stage, to a quasi-Fickian diffusion, in the second stage, corresponding to a limited diffusional transport of FA. This dual-release regime may be potentially explained by the lower stability of MLVs in suspension, which justifies a FA release associated with the destabilization/erosion of the outer layers of these vesicles. The FA release observed in the second stage might be explained by the release of FA from the more stable vesicle inner layers/vesicle core, leading to FA release governed by controlled diffusion mechanisms. Yet, this does not clearly explain the rise in the k_KP_ values in the second stage of the regime. The absence of these dual FA release regimes in liposomal hydrogels may thus be once more interpreted as the additional structural stabilization provided by liposome confinement in the hydrogel network, in agreement with that reported in further literature [25].

### 3.4. Magnetically Controlled Release of Ferulic Acid

Magnetic-responsive hydrogels, i.e., hydrogel matrices doped with magnetic susceptible components, such as the iron oxide nanoparticles (Fe_3_O_4_), are able to switch their volume and shape when exposed to a magnetic field [54,55]. Magnetic-responsive liposomal hydrogels reported in further literature have been developed for magnetic modulation of drug release based on their ability to produce hypothermia effects when exposed to an alternate magnetic field (AMF), resulting in the thermally induced release of the target drug [49,53]. In contrast, the magnetic-responsive gelatin membranes used in this work for encapsulation of DPPC:FA liposomes may also be used as mechanical actuators capable of producing mechanical forces under magnetic stimulation, which are expected to activate the release of FA from the encapsulated liposomes.

Hence, studies were conducted to evaluate the ability of the magnetic field to control the FA release from magnetic-responsive liposomal hydrogels prepared in this work. In this case, FA release assays from magnetic-responsive gelatin membranes doped with 0.25% and 1% MNPs and embedded 3:1 and 10:1 DPPC:FA liposomes were performed in the absence (reference condition) and presence of an external permanent magnetic field with an intensity of 0.08 T, produced by a neodymium magnet. 

Figure 7 shows the FA release profiles obtained for the magnetic-responsive liposomal gelatin membranes exposed and non-exposed to a magnetic field. A comparative analysis of the FA release profiles immediately evidences the effect of the liposome formulation and the %MNP present in the hydrogel membrane on the FA release. The results showed a consistently higher cumulative release of FA, varying between 22.1% and 36.8%, after 48 h for liposomal hydrogels embedded with 10:1 DPPC:FA liposomes (blue symbols), confirming the lower ability of 10:1 DPPC:FA liposomal hydrogel systems to retain the FA molecules.

Furthermore, the results show a higher FA release when the %MNP was increased from 0.25% to 1%, easily perceptible by comparing the filled (0.25 % MNPs) and empty (1% MNPs) symbols in Figure 7, evidencing that MNPs influence the delivery of FA even in the absence of a magnetic field. This effect cannot be explained by differences in the hydrogel swelling capacity, since, as discussed before, the swelling ratio obtained with gelatins with 0.25% MNPs was higher than that obtained with gelatin with a higher MNP concentration (Figure 4). However, it might be due to an additional instability of the FA molecules in the hydrogel matrix resulting from the increased polarity of the hydrogel matrix after increasing the %MNP, associated with the high hygroscopic properties of the MNPs.

**Figure A5:**
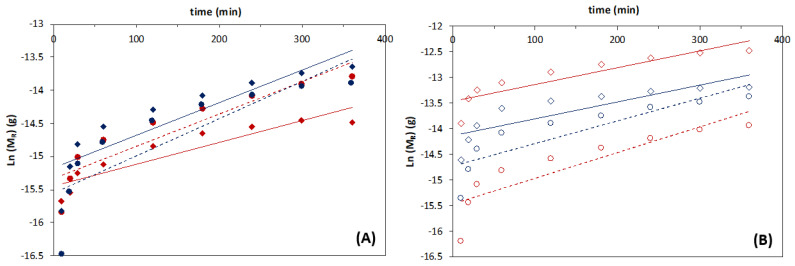
Logarithmic function of the ferulic acid (FA) mass release profiles obtained with magnetic−responsive liposomal gelatins containing (**A**) 0.25% and (**B**) 1% MNPs, with encapsulated 3:1 (red symbols) and 10:1 (blue symbols) DPPC:FA liposomes in the absence (circles) and presence (diamonds) of a magnetic field with an intensity of 0.08 T, at 37 °C, fit to a 1st-order kinetic model.

**Figure A6:**
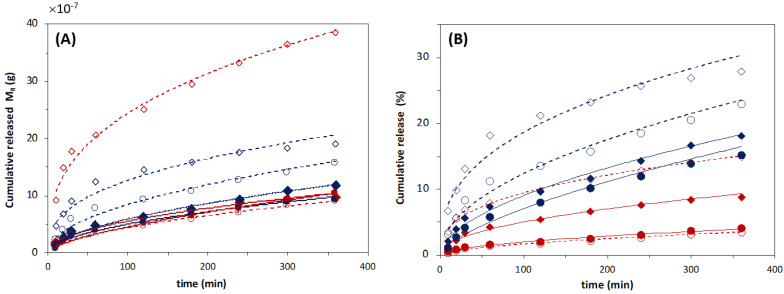
Ferulic acid (FA) release profiles obtained with a 3:1 (red symbols) and 10:1 (blue symbols) DPPC:FA liposomes encapsulated in magnetic−responsive gelatin membranes containing 0.25% MNPs (filled symbols) and 1% MNPs (empty symbols), in the absence (circles) and presence (diamonds) of magnetic field (MF) with an intensity of 0.08 T, for a period of 360 min, at 37 °C, fit to (**A**) Higuchi model, which considers the FA mass release, M_R_, and (**B**) Korsmeyer–Peppas model which considers the FA release profiles determined by M_R_/M_T_ × 100.

The effect of hydrogel formulation on the FA release kinetics and mechanisms was accessed by comparative analysis of the releasing parameters obtained by adjustment of different release kinetic models. The experimental FA release profiles are clearly not described by a zero-order kinetic model since there is not a linear increase in the cumulative mass of the released FA over time. The FA release profiles are also not properly described by first-order kinetic functions, as expressed by the low R^2^ values < 0.85 (Table A4) obtained for all delivery systems, denoting a poor fitting quality in each case (Figure A5, in Appendix A). In contrast, the FA release from these magnetic-responsive liposomal hydrogels was perfectly adjusted to Higuchi models (Figure A6A in Appendix A), with R^2^ > 0.980 in most cases (Table 5), as well as to Korsmeyer–Peppas models, as evidenced by fittings to the Korsmeyer–Peppas function shown in Figure A6B and confirmed by the resultant R^2^ values > 0.960 (Table 5).

As shown in Table 5, according to both Higuchi and Korsmeyer–Peppas models, for liposomal hydrogel delivery systems a decrease in the FA fraction from 3:1 to 10:1 DPPC:FA and an increase in the MNP content from 0.25% to 1% led a significant increase in the FA release rates, with K_H_ and K_KP_ release constants registering an increase >50% (with the only exception of the liposomal hydrogels formed with 3:1 DPPC:FA liposomes). Besides the changes in the FA release rates, the FA release showed a good adjustment to the Higuchi model, leading to exponent values in good agreement with the characteristic Higuchi model exponent, i.e., 0.5 (Table 5), which evidences a diffusional controlled release of FA in the absence of magnetic field. An identical conclusion was taken from the analysis with Korsmeyer–Peppas models. This model assumes variable n values dependent on the FA release mechanism, but as shown in Table 5, Korsmeyer-–Peppas led to n values ~0.5, quite similar to those estimated with Higuchi models, confirming a Fickian diffusion delivery of FA in the absence of a magnetic field.

It was interesting to see the effect of the magnetic field on the FA release mechanisms. In this case, the magnetically induced FA release profiles were also perfectly fit to the Higuchi model but with a significant decrease in the diffusion exponential parameter to values lower than 0.5, which suggests that despite the good fitting quality, the magnetic field stimuli shift the FA from a Higuchian model. For this reason, the effect of the magnetic field on the FA release was only evaluated based on the releasing parameters estimated by the Korsmeyer–Peppas model.

In fact, the presence of a magnetic field resulted in a remarkable increase in the FA release rates, with k_KP_ registering values more than two-fold higher than that obtained for the same liposomal hydrogel delivery system in the absence of a magnetic field. It is important to note that the increase in FA release due to the thermal effect due to the magnetic field was excluded. The presence of thermal effects is mainly associated with the use of an alternate magnetic field (AMF) and not with permanent magnetic field conditions, such as those used in the release assays described in this work. Despite this, the absence of thermal effects on magnetic-responsive polymeric systems when exposed to a magnetic field up to 1.5 T was investigated and confirmed in previous works from the same authors [56,65]. The transport of FA from the liposomal hydrogel membrane under magnetic stimulation was kept in the controlled diffusion regime, with n < 0.5. However, the magnetic stimulation induced a decrease in the n values (n < 0.4 for liposomal hydrogel systems doped with 1% MNPs), suggesting a change from a Fickian diffusional to a quasi-diffusion mechanism. This change was clearer in liposomal hydrogels containing higher %MNPs, revealing, as expected, a more accentuated effect of the magnetic stimuli in hydrogels with higher MNP concentrations. 

In analogy to the dual-release regime observed for the FA release profiles from free MLV suspensions, it might be plausible to think that the low FA release observed in the 48 h experiment in the absence of a magnetic field corresponds mainly to the release of FA located on the outer layers of the encapsulated MLVs. When the magnetic field is applied, it might force the release of FA from the internal layers or the core of MLVs by mechanical compression of the liposomes triggered by a magnetic-induced contraction of the gelatin matrix resulting from the mobility of MNPs imposed by the attraction magnetic field forces, as reported in further literature [54,55,56,57,58,59]. The magnetic-induced release may thus be associated with changes in the permeability of the liposome membrane, with or without rupture of the vesicle. Nevertheless, it might be also hypothesized that it may benefit from a lower accumulation of FA in the hydrogel matrix (due to a higher diffusion of FA from the hydrogel matrix upon magnetic hydrogel contraction), thus minimizing the potential decrease in the driving force for the FA liposomal release during the process.

Overall, these results prove that the magnetic behavior of hydrogels doped with MNPs may effectively be used for the activation of drug release, suggesting the potential use of magnetic-responsive liposomal hydrogels as delivery platforms for an enhanced controlled and on-demand delivery of therapeutic compounds/bioactive molecules. However, further work is still needed to better clarify the impact of this magnetic stimulatory strategy on the structural integrity of the encapsulated liposomes and the reproducibility of this drug release process. This work shows that magnetic-induced release can be accomplished using low magnetic field intensities (0.08 T), which is minimally invasive to the human organism, contributing to the development of magnetic-responsive drug delivery for long-term treatments and simulating its integration into tissue engineering systems which may be further explored as novel and more efficient magnetic-responsive therapeutics for tissue repair [58].

## 4. Conclusions

This work was focused on the development of magnetic-responsive liposomal hydrogel membranes while proving their ability to provide enhanced magnetically controlled release of small molecules with therapeutic relevance and poor water solubility, such as ferulic acid (FA), based on the mechanical responsive behavior (mechanical distortions) of hydrogels doped with iron oxide nanoparticles (MNPs) upon magnetic stimulation. Magnetic-responsive liposomal hydrogels were found to delay the release of FA comparatively to that observed with conventional drug release systems, e.g., MLV suspensions or native hydrogel matrices. The delayed FA release was attributable to the additional stability of entrapped multilayered DPPC vesicles loaded with FA as well as to some resistance offered by the hydrogel matrix to the FA diffusion, resulting in a more sustained and longer-term release of this therapeutic molecule. The FA release from liposomal hydrogel systems was well described by the Korsmeyer–Peppas model, which showed that FA release followed a Fickian diffusion mechanism identical to that found for native hydrogels in the absence of a magnetic field. However, the encapsulation of MLVs into the hydrogel matrix renders a remarkable decrease in the constant release rates, confirming the delayed FA release from liposomal hydrogels. These results primarily suggest the promising use of these liposomal (MLVs) hydrogel platforms in therapeutics requiring a longer-term drug administration as an alternative to the less efficient conventional drug delivery systems while evidencing the valuable utilization of MLVs in biomedicine following their inclusion into hydrogel matrices. 

The liposomal hydrogel delivery platforms were shown to be able to magnetically stimulate drug release, owing to the presence of dispersed MNPs. The magnetic-induced release of FA from these liposomal gelatin membranes was expressed by a significant increase in the release constant rates while keeping a diffusional controlled FA release mechanism but changing the FA release from Fickian diffusion to quasi-Fickian diffusion.

Magnetic-responsive liposomal hydrogels may be used as an independent non-cellular drug delivery strategy offering an on-demand and efficient magnetically controlled drug release into the target tissue, thus minimizing the drug losses caused by burst release. Furthermore, it may be easily combined with tissue engineering approaches, e.g., by integration of liposomes into magnetic-responsive tissue scaffolds. The impact of these magnetic-responsive liposomal hydrogels on the metabolic behavior of mammalian cells will be the focus of the following studies aiming to further explore and evaluate their potential for the development of novel and more efficient tissue repair strategies. On the one hand, they will take advantage of the magnetic responsiveness of these hydrogels to obtain in situ fine-tuned delivery of therapeutic agents; on the other hand, they will be used for the magnetic stimulation of key cell mechanotransduction processes for triggering improved cell development and faster tissue regeneration.

## Figures and Tables

**Table 1:** Mean sizes and the respective PI values obtained for DPPC liposomes dispersed in PBS solution with different dilution factors and exposed to different sonication times.

Liposome	Dilution Factor	Sonication Time (min)	Mean Size (nm)	PI
DPPC_5_30	5×	30	705.3 ± 155.8	0.52 ± 0.03
DPPC_10_30	10×	30	559.5 ± 27.6	0.76 ± 0.21
DPPC_5_45	5×	45	270.6 ± 139.2	0.53 ± 0.09
DPPC_10_45	10×	45	237.7 ± 65.4	0.51 ± 0.12

**Table 2:** Mean size and PI values obtained for non-diluted solutions of unloaded DPPC liposomes and DPPC liposomes loaded with 3:1, 10:1 and 30:1 DPPC:FA ratios, upon 60 min of sonication, before and after purification by dialysis.

Liposome	Purification	Mean Size (nm)	PI
DPPC	No	106.6 ± 45.9	0.36 ± 0.34
DPPC:FA_30:1		716.5 ± 103.7	1.49 ± 0.44
DPPC:FA_10:1		1385.7 ± 443.3	0.12 ± 0.01
DPPC:FA_3:1		1200.0 ± 257.8	0.12 ± 0.11
DPPC:FA_30:1	Yes	714.1 ± 152.9	0.71 ± 0.01
DPPC:FA_10:1		992.6 ± 121.0	0.15 ± 0.06
DPPC:FA_3:1		775.9 ± 39.4	0.17 ± 0.02

**Table 3:** Encapsulation efficiency (EE) and loading capacity (LC) obtained for liposomes prepared with different DPPC:FA ratios, upon purification by dialysis.

Liposome	EE (%)	LC (%)
DPPC:FA_30:1	62.1	1.28
DPPC:FA_10:1	73.53	6.73
DPPC:FA_3:1	88.06	14.01

**Table 4:** Release constant (k_KP_) and diffusion exponential (n) obtained for the FA release from gelatin membranes doped with free FA, free liposomes prepared using 3:1 and 10:1 DPPC:FA fractions and 3:1 and 10:1 DPPC:FA liposomes encapsulated in gelatin membranes over 48 h, at 37 °C.

	Hydrogel + FA	DPPC:FA Hydrogel		DPPC:FA Suspension	
		3:1	10:1	3:1	10:1
k_KP_	3.818 ± 0.022	0.264 ± 0.068	1.104 ± 0.090	0.352 ± 0.197 5.810 ± 0.059	0.238 ± 0.067 12.460 ± 0.080
n	0.428 ± 0.051	0.426 ± 0.028	0.405 ± 0.037	0.920 ± 0.129 0.223 ± 0.022	0.794 ± 0.033 0.129 ± 0.027
R^2^	0.980	0.934	0.915	0.926 0.940	0.970 0.875

**Table 5:** Release constants (k_H_ and k_KP_) and diffusion exponential (n) obtained for 3:1 and 10:1 DPPC:FA liposomal gelatin systems for a period of 360 min in the presence and absence of a 0.08 T magnetic field, at 37 °C.

Liposomal Hydrogel		0.25% MNPs				1% MNPs			
		3:1	3:1_MF	10:1	10:1_MF	3:1	3:1_MF	10:1	10:1_MF
Higuchi	k_H_ (×10^−7^)	0.225 ± 0.107	0.567 ± 0.042	0.428 ± 0.044	0.464 ± 0.064	0.331 ± 0.080	4.594 ± 0.043	0.837 ± 0.073	2.193 ± 0.057
	n = 0.5	0.651 ± 0.054	0.494 ± 0.021	0.531 ± 0.022	0.551 ± 0.032	0.562 ± 0.040	0.363 ± 0.022	0.501 ± 0.036	0.381 ± 0.029
	R^2^	0.983	0.988	0.989	0.991	0.988	0.990	0.984	0.966
Korsmeyer–Peppas	k_KP_	0.174 ± 0.044	0.550 ± 0.055	0.333 ± 0.103	0.715 ± 0.064	0.129 ± 0.079	1.784 ± 0.043	1.235 ± 0.073	3.237 ± 0.057
	n	0.531 ± 0.022	0.481 ± 0.028	0.663 ± 0.052	0.551 ± 0.032	0.562 ± 0.040	0.363 ± 0.022	0.501 ± 0.036	0.381 ± 0.029
	R^2^	0.989	0.990	0.990	0.991	0.988	0.990	0.984	0.966

## Data Availability

Experimental data are available upon request.

## Appendix A

**Table A1:** Mean size and PI values obtained for DPPC liposomes loaded with 30:1 and 10:1 DPPC:FA ratios, upon 45 min of sonication, before and after purification by dialysis.

Liposome	Purification	Mean Size (nm)	PI
DPPC:FA_30:1 DPPC:FA_10:1	No	716.5 ± 103.7 1930.1 ± 101.7	1.49 ± 0.44 0.63 ± 0.09
DPPC:FA_30:1 DPPC:FA_10:1	Yes	714.1 ± 152.9 998.5 ± 131.0	0.71 ± 0.01 0.63 ± 0.05

**Table A2:** Release constant k_1_ (min^−1^) obtained by fitting of the 1st-order kinetic function to the FA release from magnetic-responsive gelatin membranes with embedded FA and from liposome suspensions with 10:1 and 3:1 DPPC:FA fractions at 37 °C.

	Hydrogel + FA	DPPC:FA Suspension	
		3:1	10:1
k_1_ (min^−1^)	1.6 × 10^−3^	3.4 × 10^−3^	7.1 × 10^−3^
R^2^	0.9205	0.767	0.933

**Table A3:** Higuchi constant, k_H_, and diffusion exponential, obtained by fitting of Higuchi model to the FA release from magnetic-responsive gelatin membranes with embedded FA and from liposome suspensions with 10:1 and 3:1 DPPC:FA fractions at 37 °C.

	Hydrogel + FA	DPPC:FA Suspension	
		3:1	10:1
k_H_	44.1 × 10^−7^	2.4 × 10^−7^	10.2 × 10^−7^
n = 0.5	0.641	0.807	0.141
R^2^	0.945	0.968	0.983

**Table A4:** First-order rate constant, k_1_ (min^−1^), and R^2^ values obtained by fitting of first-order kinetic function to the FA release profiles from magnetic-responsive liposomal gelatins containing 0.25% and 1% MNPs and encapsulated 3:1 and 10:1 DPPC:FA liposomes in the absence and presence of a magnetic field with an intensity of 0.08 T, at 37 °C.

	0.25% MNPs				1% MNPs			
	3:1	10:1	3:1 MF	10:1 MF	3:1	10:1	3:1 MF	10:1 MF
k_1_ (min^−1^)	0.0049	0.0056	0.0033	0.0049	0.0050	0.0044	0.0033	0.0032
R^2^	0.719	0.847	0.797	0.844	0.777	0.753	0.8035	0.7128
